# Molecular Mechanisms of the Cytotoxic Effect of Recombinant Selenoprotein SELENOM on Human Glioblastoma Cells

**DOI:** 10.3390/ijms24076469

**Published:** 2023-03-30

**Authors:** Vladimir V. Rogachev, Michael V. Goltyaev, Elena G. Varlamova, Egor A. Turovsky

**Affiliations:** Institute of Cell Biophysics of the Russian Academy of Sciences, Federal Research Center “Pushchino Scientific Center for Biological Research of the Russian Academy of Sciences”, 142290 Pushchino, Russia

**Keywords:** selenium, selenoprotein SELENOM, cancer, glioblastoma

## Abstract

Currently, selenobiology is an actively developing area, primarily due to the study of the role of the trace element selenium and its organic and inorganic compounds in the regulation of vital processes occurring in the cell. In particular, the study of the functions of selenium nanoparticles has gained great popularity in recent years. However, a weak point in this area of biology is the study of the functions of selenoproteins, of which 25 have been identified in mammals to date. First of all, this is due to the difficulties in obtaining native forms of selenoproteins in preparative quantities, due to the fact that the amino acid selenocysteine is encoded by one of the three stop codons of the TGA universal genetic code. A complex system for recognizing a given codon as a selenocysteine codon has a number of features in pro- and eukaryotes. The selenoprotein SELENOM is one of the least studied mammalian selenoproteins. In this work, for the first time, studies of the molecular mechanisms of regulation of the cytotoxic effect of this protein on human glioblastoma cells were carried out. The cytotoxicity of cancer cells in our experiments was already observed when cells were exposed to 50 μg of SELENOM and increased in proportion to the increase in protein concentration. Apoptosis of human glioblastoma cells was accompanied by an increase in mRNA expression of a number of pro-apoptotic genes, an increase in endoplasmic reticulum stress, and activation of the UPR IRE1α signaling pathway. The results obtained also demonstrate a dose-dependent depletion of the Ca^2+^ pool under the action of SELENOM, which proves the important role of this protein in the regulation of calcium homeostasis in the cell.

## 1. Introduction

Selenium (Se) is a biologically active trace element that is part of a number of important enzymes in the form of the amino acid selenocysteine, and is thus associated with all organs and systems. To date, 25 human selenium proteins have been identified, while murine and rat selenoproteomes are represented by 24 proteins. Most of them can be classified into two groups based on the localization of the selenocysteine residues [1]. The first group includes selenoproteins characterized by the presence of selenocysteine in the C-terminal sequence of these proteins. Thus, in mammalian thioredoxin reductases containing the GCUG motif, selenocysteine is localized in the mobile C-terminal position [2], which allows the catalytic center to transfer reduced equivalents from the hard-to-reach disulfide active site to the active site of the substrate protein.

The second selenoprotein group includes proteins containing selenocysteine at the N-terminal position of the short domains. This is a large group of α/β proteins in which, according to their secondary structure, the selenocysteine-containing catalytic center is located between the α-helix and the β-sheet, similar to the arrangement of the CXXC motif (sequence in which two cysteines are separated by two other amino acids) of oxidoreductases, involved in thiol-disulfide exchange. Selenocysteine in the redox center can replace both N- and C-terminal cysteine with the formation of CXXU/UXXC motifs [3]. This group of proteins also includes SELENOM, a highly conserved protein expressed in many tissues, but mostly in the brain [4].

According to its structural features, the protein, along with SELENOT and SELENOF, belongs to the family of proteins with a thioredoxin-like fold. That is, the location of the elements of the secondary structure in these proteins is close to thioredoxin [5]. SELENOM can be considered a marker of brain selenium deficiency, as it is sensitive to changes in the concentration of this trace element in the brain. In addition, this selenoprotein is known to be involved in the regulation of a number of neurodegenerative diseases [6]. Using HT22 hippocampal cells and C8-D1A cerebellar cells as an example, SELENOM was shown to be involved in preventing hydrogen peroxide-induced oxidative damage to cells [7]. Knockdown of the SELENOM gene in rats resulted in an increase in the activity of glutathione peroxidases and thioredoxin reductases in the brain, liver, lungs, and, to a lesser extent, in the kidneys and heart. In addition, it is known that SELENOM may be involved in the regulation of calcium homeostasis, which has been demonstrated in HT22 and C8-D1A cancer cells. This selenoprotein increased the concentration of cytosolic calcium in response to oxidative stress and thus participated in the regulation of apoptosis, blocking it [8]. To date, there are few studies on the role of SELENOM in carcinogenesis, with the first data on such regulation were shown with the example of hepatocellular carcinoma (HCC) cells [9].

Previously, we selected the optimal conditions for the expression of recombinant human SELENOM, in the open frame of which selenocysteine (Sec) was replaced by cysteine (Cys). Protein preparation in preparative amounts was carried out in a bacterial system [10]. Using affinity chromatography in tandem with mass spectrometric analysis using MALDI–TOF, we previously identified two isoforms of cytoplasmic actin as possible physiological partners of SELENOM in MCF–7 and HT–1080 cells: cytoskeletal β-actin and cytoskeletal γ-actin [10]. These two main isoforms of cytoplasmic actin differ from each other by only four amino acid residues at the N-terminus and play a key role in key cellular processes such as adhesion, migration, polarization, and mitosis. During tumor transformation, pathological changes in cell motility occur, caused by dysregulation of the actin system, which leads to tumor invasion and metastasis.

It is known that another selenoprotein of the thioredoxin-like family, SELENOF, is a structural homologue of SELENOM and is also localized in the ER [11]. Interestingly, a decrease in SELENOF activity in various cancer cells led to plasma membrane blebbing [12], a process in which actin plays an important role: actin microfilaments are reorganized into membrane-bound peripheral annular bundles, while the cell acquires a rounded shape and further contraction of the rings leads to membrane blebbing and ultimately to cell apoptosis. This may indicate that SELENOF is functionally associated with actin filaments and is involved in the regulation of the cytoskeleton in the cell. Since SELENOM is structural homologue of SELENOF, that’s why it may be also involved in the regulation of similar processes in human cancer cell lines; however, this assumption requires further confirmation using other independent approaches.

In addition, we have previously shown that SELENOM-KD in human glioblastoma cells (A-172 line) leads to increased expression of a number of key pro-apoptotic genes, two selenoproteins SELENOT and SELENOK, and two glutathione peroxidases GPX1 and 2, thioredoxin reductase 3 (TXNRD3), as well as the transcription factor ATF-4, which may indicate activation of the PERK signaling pathway UPR [13]. In addition, it was shown that a decrease in SELENOM activity in glioblastoma cells contributed to the functional disorders of the ER, reducing its ability to deposit Ca^2+^ ions, which led to the emptying of the ER capacity. Most likely, such an effect on the ER can significantly change the expression of proteins that regulate the pathways of cell viability.

The aim of this work was to study the molecular mechanisms of the cytotoxic effect of recombinant human SELENOM (hSELENOM) on human glioblastoma cells (A-172 line). This study will certainly complement the existing data regarding the functions of this selenoprotein and its role in carcinogenesis.

## 2. Results

### 2.1. Optimal Conditions for the Synthesis of Human SELENOM in a Bacterial System Were Observed When Bacterial Cells Were Incubated with 0.5 MM IPTG for 3 h at 25 °C

When studying the functions of any protein, it is necessary to obtain it in preparative quantities, which is often a difficult task. This is especially true in cases where it is necessary to express the gene of a eukaryotic protein in a bacterial system. In the case of selenoproteins, this task is complicated by the fact that the amino acid selenocysteine (Sec), which is part of them, is encoded by one of the three stop codons of translation. In order for it to be recognized as selenocysteine and not as a premature termination of translation, as a rule, many authors work with cysteine homologues of selenoproteins. In rare cases, it is possible to choose conditions for the synthesis of native forms of selenoproteins in preparative amounts, which is explained by the peculiarities of recognition of the selenocysteine codon and differences in cis- and trans-active factors in bacterial and eukaryotic systems [14,15].

Despite the fact that earlier we were able to select the conditions for the production of the cysteine homologue hSELENOM, in the framework of this work we further improved them, which allowed us to obtain an even higher yield of purified protein (up to 10 mg/mL), taking into account the fact that this protein tends to form inclusion bodies.

**Figure 1:**
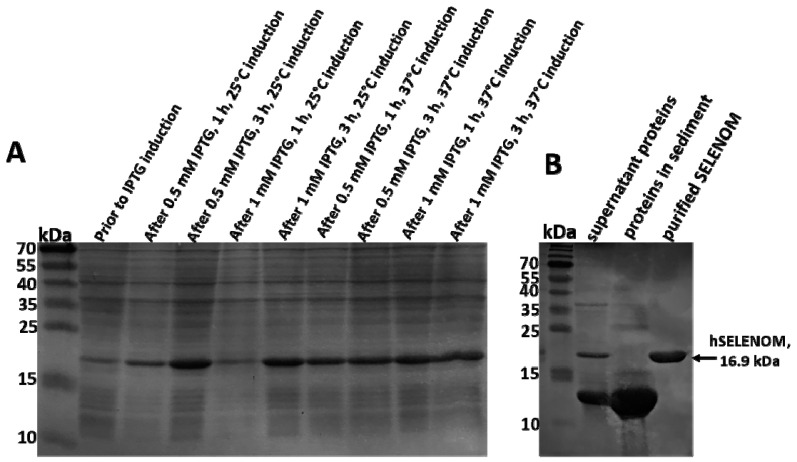
PAGE-electrophoresis of samples of different stages of hSELENOM ORF induction in bacterial cells. (**A**) Expression of hSELENOM under various conditions in bacterial cells; (**B**) PAGE-electrophoresis of proteins in the soluble fraction, sediment, and purified hSELENOM on nickel agarose. PAGE-electrophoresis was performed using 12.5% acrylamide gels.

Figure 1A shows the results of the selection of optimal conditions for achieving high expression of the open reading frame (ORF) of the gene encoding the cysteine homologue of hSELENOM. Based on the above data, it can be concluded that the maximum level of expression of the ORF gene encoding the human cysteine homologue SELENOM (TGA– > TGT) in the pET 23b (+) plasmid was observed when bacterial cells were treated with 1 mM IPTG for 3 h at 25 °C and 1 mM IPTG for 1 h at 37 °C. However, when cells were incubated with 0.5 mM IPTG for 3 h at 25 °C, only approximately 20–30% of the total protein precipitated, with most remaining in the soluble phase. Therefore, in our case, the optimal conditions for the synthesis of recombinant hSELENOM in bacterial cells of the Rosetta (DE3)pLysS strain (Novagen, USA) were achieved by inducing the lactose operon with 0.5 mM IPTG for 3 h at 25 °C. Figure 1B shows the results of PAGE electrophoresis visualizing the various stages of protein purification under study.

### 2.2. Recombinant hSELENOM Reduced Proliferative Activity and Induced Apoptosis in Human Glioblastoma Cells

**Figure 2:**
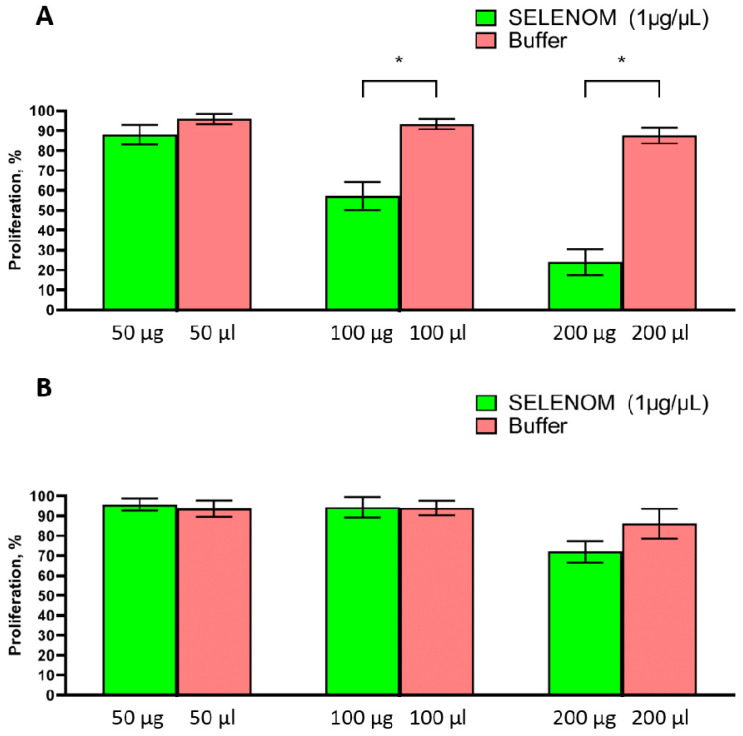
Analysis of the proliferative activity of cancer cells A-172 (**A**) and embryonic kidney cells HEK 293 (**B**) after 24 h exposure to different amounts of recombinant hSELENOM (1 µg/µL). Optical density was measured at 590 nm and the values of the corresponding untreated cells were taken as 100%. Green columns correspond to the values of the proliferative activity of the cells of the two studied lines, incubated in medium containing 50, 100, or 200 µg hSELENOM (1 µg/µL). The pink columns correspond to the values of the proliferative activity of the cells of the two studied lines, incubated in medium containing 50, 100, or 200 µL of buffer. Mean values ± standard errors (SE) were determined by analyzing data from at least three independent experiments; * *p* < 0.05.

On Figure 2A shows the results of MTT analysis of the proliferative activity of human glioblastoma cells (A-172 lines) after a 24 h incubation in a nutrient medium supplemented with various amounts (50, 100, and 200 µg) of hSELENOM. The seeding density of cells in the experimental groups was the same, and hSELENOM and buffer application were made 2 days after cell seeding. At this time, the cells are in the active stage of proliferation. According to the presented results, it can be concluded that recombinant hSELENOM was able to reduce the proliferative activity of A-172 cancer cells in direct proportion to the increase in the concentrations of this protein in the nutrient medium. As a control, cells were incubated under the same conditions, but with the addition of protein elution buffer. According to Figure 2A, it can be concluded that a significant decrease (by approximately 40%) in the proliferative activity of glioblastoma cells was observed after exposure of A-172 cells to 100 μg of protein, while a doubling of the protein concentration contributed to a decrease in the proliferative activity of cells by approximately 70–75%.

**Figure 3:**
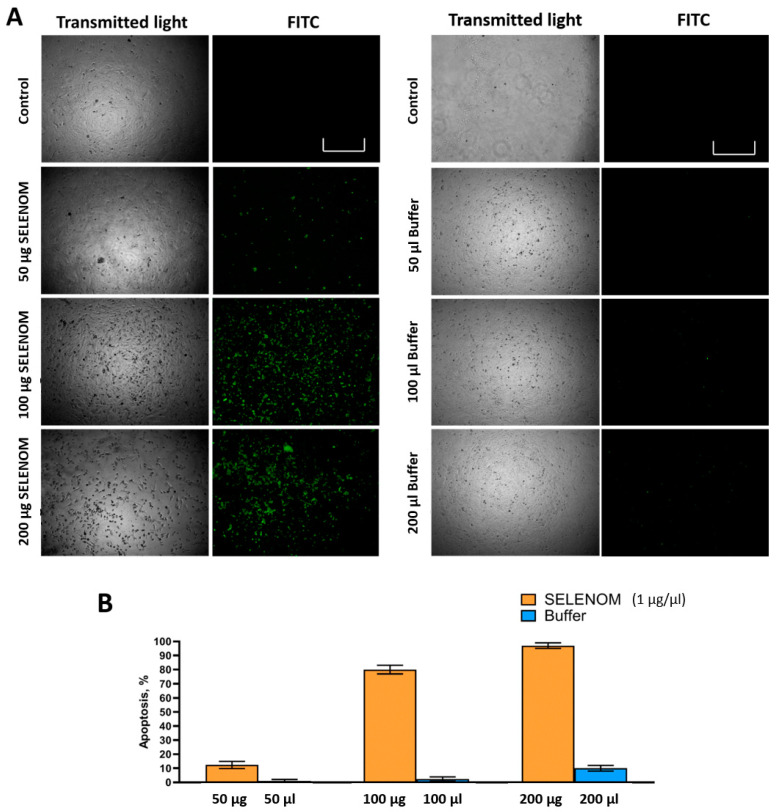
Visual assessment of A-172 cells in which apoptotic death is activated or not activated after 24 h of exposure to various amounts of recombinant hSELENOM (1 µg/µL). (**A**) Visual assessment of the state of A-172 cells in transmitted light and using fluorescence microscopy. Green dye shows cells with signs of apoptosis. Scale bar–500 µm (**B**) Quantification of cells with and without apoptosis. Mean values ± standard errors (SE) were determined by analyzing data from at least three independent experiments.

Figure 3A,B show the results of the analysis of the presence of signs of apoptotic death of glioblastoma cells. According to the data obtained, it can be concluded that hSELENOM was able to induce apoptosis in glioblastoma cells. The number of apoptotic cells increased with increasing protein concentration in the nutrient medium. According to the results presented in Figure 3B, we can confidently say that almost 70% of the cells were in a state of apoptotic death when A-172 cells are treated with 100 μg hSELENOM. Doubling the amount of protein enhanced the cytotoxic effect and the number of apoptotic cells increased up to almost 90% compared with the control.

### 2.3. Recombinant hSELENOM Induced Increased mRNA Expression of Pro-Apoptotic Genes and the IRE1α Signaling Pathway UPR

**Figure 4:**
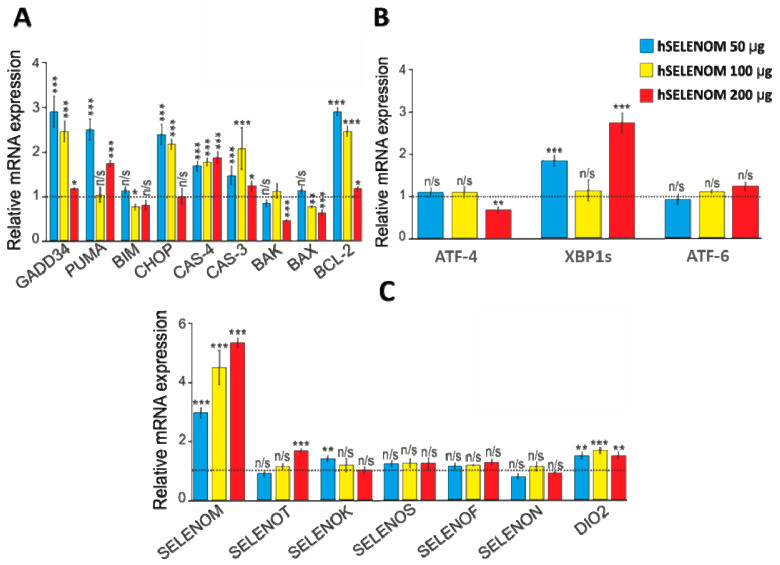
Analysis of mRNA expression of apoptotic genes (**A**), ER stress-associated genes (**B**), and genes encoding selenoproteins (**C**) in A-172 cells after 24 h exposure to recombinant hSELENOM (1 µg/µL). The control in these experiments was the analysis of mRNA expression of the studied genes in A-172 cells after 24 h of exposure to the buffer. The control is taken as 1 and is shown as a dotted line. Blue bars show mRNA expression levels of the studied genes after cell treatment with 50 µg hSELENOM, yellow bars after treatment with 100 µg hSELENOM, and red bars after treatment with 200 µg hSELENOM. Mean values ± standard errors (SE) were determined by analyzing data from at least 3 independent experiments; n/s—data are not significant (*p* > 0.05), * *p* < 0.05, ** *p* < 0.01, *** *p* < 0.001.

The above results were confirmed by the analysis of mRNA expression patterns for a number of pro-apoptotic genes. Thus, 24 h exposure of human glioblastoma cells to various amounts of hSELENOM led to the increased expression of a number of pro-apoptotic genes by two or more times (Figure 4A). Interestingly, lower amounts of this protein (50 and 100 μg) contributed to the greatest increase in the expression of these genes, while 200 μg of the protein did not significantly affect their expression.

Increased mRNA expression of genes such as CHOP, GADD34, PUMA, and CAS-4 may also indicate the activation of endoplasmic reticulum stress (ER stress) in these cells. In addition, when analyzing the mRNA expression patterns shown in Figure 4A, it is easy to see that 50 and 100 µg hSELENOM caused an increase in the expression of the anti-apoptotic BCL-2 gene, which was not observed when cells were exposed to 200 µg hSELENOM.

**Figure 5:**
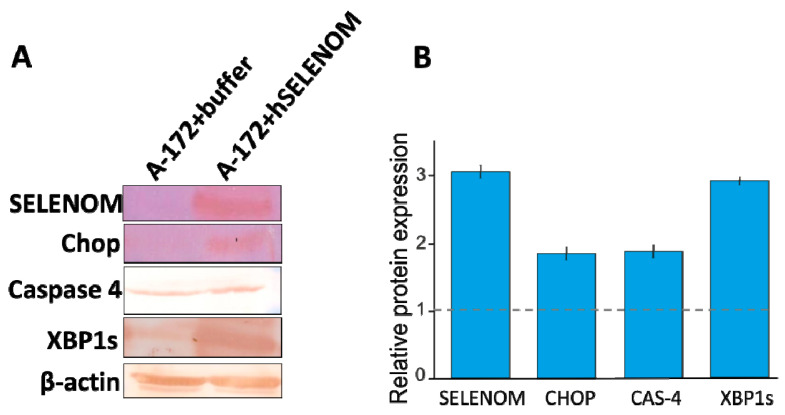
Quantification of proteins by Western blotting of A-172 cells after 24 h of exposure to 100 μg of recombinant hSELENOM (1 μg/μL) in Supplementary Materials. Samples of A-172 cells treated with the appropriate volume of buffer (100 µL) served as controls in these experiments. The control is taken as 1 and is shown in the figure as a dotted line. (**A**) Relative content of proteins (endogenous selenoprotein SELENOM, Chop, Caspase-4, and XPB1s) in cells and visualization of the results obtained using Western blot. (**B**) Quantitative assessment of proteins in the studied samples in comparison with the control.

Direct evidence of increased ER stress was also provided by the fact that this protein promoted an increase in the expression of a marker of one of the three signaling pathways of the cell’s UPR response to the growth of misfolded proteins. Therefore, according to the results shown in Figure 4B, there was an increase in the expression of the spliced form of the XBP1s transcription factor. This may indicate the activation of the IRE1α signaling pathway UPR. These data were confirmed by Western blotting, the results of which are shown in Figure 5A,B.

In addition, we analyzed how the expression of seven ER-resident selenoproteins changed, since their role in the regulation of apoptosis, ER stress, post-translational modification of proteins in the ER, and calcium homeostasis has been repeatedly proven [16,17,18,19]. According to the real-time PCR results shown in Figure 4C, we can confidently speak about a significant increase in the mRNA of the endogenous selenoprotein SELENOM by more than 3–5 times compared with the control when treating human glioblastoma cells with 50, 100, and 200 μg of recombinant hSELENOM. At the same time, we did not observe an increase in mRNA expression of the genes of the other six ER proteins.

### 2.4. Recombinant hSELENOM Depleted Endoplasmic Reticulum Ca^2+^ Pools

**Figure 6:**
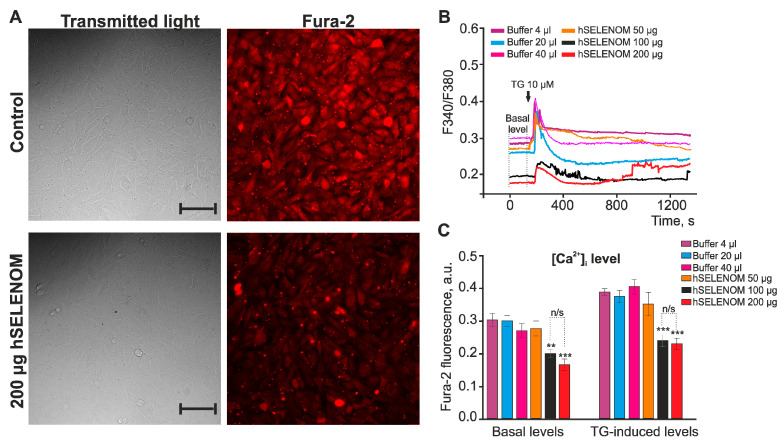
Effect of recombinant hSELENOM on the Ca^2+^ signaling system of human glioblastoma A-172 cells. (**A**) Images of A-172 human glioblastoma cell culture in transmitted light and fluorescence detection channel (380 nm) of calcium-sensitive probe Fura-2 after 24 h treatment with 200 µg hSELENOM and an equivalent volume of solvent buffer (40 µL). Scale bar–100 µm (**B**) Baseline level of [Ca^2+^]_i_ of A-172 human glioblastoma cells after application of 10 μM thapsigargin (TG) in a calcium-free medium supplemented with 0.5 mM EGTA, incubated with various amounts of hSELENOM or an equivalent volume of diluent buffer. Cellular Ca^2+^ signals averaged over 100 cells for each curve in one experiment are shown. (**C**) Averaged values of baseline [Ca^2+^]_i_ and [Ca^2+^]_i_ levels after addition of TG (10 µM) in calcium-free medium and incubated for 24 h with different concentrations of hSELENOM. Data shown are the mean value ± SE of the [Ca^2+^]_i_ levels obtained from several hundred cells. Number of passages = 3; n/s—data not significant (*p* > 0.05), ** *p* < 0.01, *** *p* < 0.001.

To study the effects of exogenous hSELENOM on the Ca^2+^ signaling system of cells, the cultures were loaded with the calcium-sensitive Fura-2AM probe and, using fluorescence microscopy, changes in the baseline and thapsigargin-induced changes in the levels of cytosolic calcium ([Ca^2+^]_i_) were recorded after a 24 h incubation with different amounts of hSELENOM. After 24 h of incubation of A-172 cells with 200 μg hSELENOM or an equivalent amount of buffer, there was no significant change in the number and morphology of cells recorded by light microscopy. At the same time, cells in the early stages of apoptosis when their morphology has not yet changed, retained the integrity of the cell membrane. However, the Fura-2 fluorescence levels in the 380 nm detection channel was significantly lower, which may indicate a lower base level of [Ca^2+^]_i_ (Figure 6A).

The analysis of the images of cells loaded with Fura-2 showed that the baseline level of [Ca^2+^]_i_ in A-172 cells after 24 h incubation with different volumes of buffer did not change (Figure 6B—Basal level) and, on average, was significantly higher compared to cells preincubated with hSELENOM at concentrations of 100 and 200 μg (Figure 6C). However, after 24 h of pre-incubation of cells with hSELENOM (100 and 200 μg), a lower level of Fura-2 fluorescence was detected, which may reflect the baseline level of [Ca^2+^]_i_ (Figure 6B,C).

**Figure 7:**
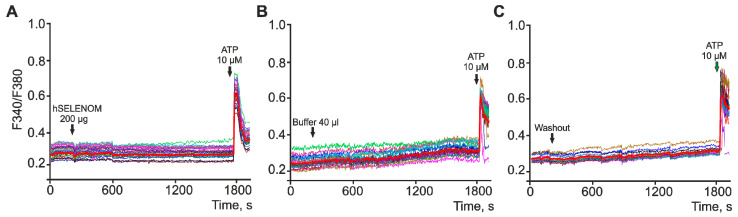
Ca^2+^ signals in A-172 human glioblastoma cells after application of 200 µg hSELENOM (**A**), solvent buffer (**B**), after washing with HBSS (**C**) and addition of ATP (10 µM). Ca^2+^ signals of cells in one experiment and their average value are shown (red bold curve). The experiments were performed in 3 repetitions on cell cultures from different passages.

The results presented above showed the dose-dependent effect of hSELENOM on the Ca^2+^ signaling system of cancer cells, which was observed during a long 24 h incubation of the cells with the protein. Application of SELENOM 200 µg to A-172 cells in an acute experiment did not cause cellular Ca^2+^ responses (Figure 7A), including no increase in the baseline level of [Ca^2+^]_i_ during the 30 min registration of Fura-2 fluorescence. In the control sample, the application of buffer (Figure 7B) also did not cause Ca^2+^ signaling in the cells. Ca^2+^ responses to ATP application were, on average, similar in all three experimental groups and close to control cells (Figure 7C), which were washed out with the working medium (HBSS).

### 2.5. Activation of the Mechanism of Actin-Dependent Endocytosis under the Action of Recombinant hSELENOM on Human Glioblastoma Cells

Since the effects of recombinant hSELENOM on A-172 cancer cells were realized within a day, it can be assumed that the protein needs to penetrate into the cells, which takes time and probably activates the mechanisms of endocytosis. To test this hypothesis, cells were exposed to actin-dependent endocytosis inhibitor Cytochalasin D (Cyt D, 10 μM) for 2 h, after which 200 μg hSELENOM was added for 24 h (with Cytochalasin D still in the culture medium). It is known that Cytochalasin D blocks or largely suppresses all actin-dependent pathways of endocytosis, depending on the duration of incubation (from 2 to 24 h) [20]. 

**Figure 8:**
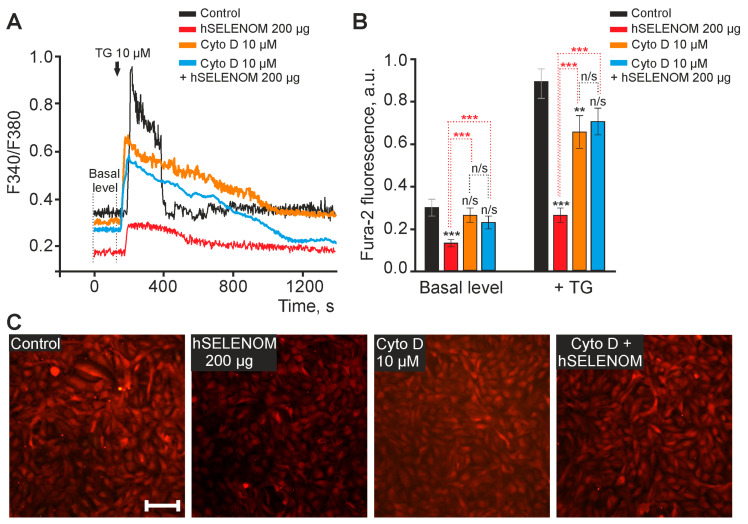
The mechanism of actin-dependent endocytosis is involved in the effects of recombinant hSELENOM on baseline and thapsigargin-induced [Ca^2+^]_i_ levels in A-172 cells. (**A**) Basal [Ca^2+^]_i_ levels and Ca^2+^ signals of A-172 human glioblastoma cells in response to the application of 10 μM thapsigargin (TG) in calcium-free medium containing 0.5 mM EGTA in control, after 24 h incubation of cells with 200 μg hSELENOM, the actin-dependent endocytosis blocker Cytochalasin D (Cyto D, 10 μM, 24 pre-incubation), and Cytochalasin D together with hSELENOM (Cyto D, 10 μM + SELENOM, 200 μg; 24 h pre-incubation). Ca^2+^ signals averaged over 100 cells for each curve in one experiment are shown. (**B**) Mean values of baseline [Ca^2+^]_i_ (Basal level) and thapsigargin-induced increase in [Ca^2+^]_i_ (+ TG, 10 μM) in calcium-free medium in control or 24 h incubation with hSELENOM, Cytochalasin D, or hSELENOM together with Cytochalasin D. Mean values ± SE of the [Ca^2+^]_i_ level obtained from several hundred cells for each column are shown. Number of passages = 3. Statistical comparisons made vs. control are marked with black asterisks. Statistical comparisons regarding hSELENOM are indicated by red asterisks. n/s—data not significant (*p* > 0.05), ** *p* < 0.01, *** *p* < 0.001. (**C**) images of A-172 human glioblastoma cell culture in fluorescence detection channel (380 nm) of calcium-sensitive probe Fura-2 in control, after 24 h treatment with 200 µg hSELENOM, Cytochalasin D (Cyto D, 10 μM, 24 h pre-incubation), and 200 µg hSELENOM together with 10 μM Cytochalasin D. Scale bar–100 µm.

After the addition of hSELENOM to the medium, a decrease in the baseline level of Fura-2 fluorescence by 50% was observed, and the TG-induced signal was reduced by 70%, relative to the control (Figure 8B). Pre-incubation of A-172 cells with Cytochalasin D for 24 h did not significantly affect the baseline level of [Ca^2+^]_i_ (Figure 8A,B), but caused a 30% decrease in the Ca^2+^ signal of cells after the addition of TG, relative to the control (Figure 8B). At the same time, the use of hSELENOM in the background of Cytochalasin D led to the similar levels Fura-2 fluorescence as cells incubated with Cytochalasin D without hSELENOM. The TG-induced increase in [Ca^2+^]_i,_ on average, also did not change significantly, which indicates the activation of the mechanisms of actin-dependent endocytosis under the action of recombinant hSELENOM in human glioblastoma cells.

## 3. Discussion

It is known that selenium and selenium-containing organic and inorganic compounds have a cytotoxic effect on various cancer cells [21,22,23,24,25,26]. Relatively recently, a similar cytotoxic effect of selenium nanoparticles was demonstrated [18,27,28,29,30,31,32]. However, selenium-containing proteins still play an important role, as this trace element is part of the active catalytic centers in the form of the amino acid selenocysteine. Despite the fact that 25 mammalian selenoproteins were identified several decades ago [33], the functions of most of them, namely their role in carcinogenesis, are still poorly understood [19,34,35,36,37,38,39,40]. These selenoproteins include SELENOM.

It is known that SELENOM is expressed in many tissues, but to a greater extent in the brain [4], is sensitive to selenium deficiency in the brain and can serve as a molecular biomarker of selenium status in this organ, and is involved in the regulation of human neurogenerative diseases [6]. Similar results were shown in HT22 hippocampal and C8-D1A cerebellar cells: stable overexpression of SELENOM prevented hydrogen peroxide-induced oxidative damage to cells [7]. According to the available data, it becomes clear that SELENOM is important for normal brain development, and its expression in various brain cancer cells is quite high. Previously, we conducted studies on this cell line under hSELENOM-KD conditions; therefore, the purpose of this work was to investigate the role of exogenous hSELENOM on the same line of human cancer cells and to conduct a comparative analysis of the regulation mechanisms of key processes occurring in cancer cells and controlling their cytotoxicity.

In this work, it was shown that the incubation of A-172 cells in a nutrient medium containing recombinant hSELENOM for 24 h led to a decrease in the proliferative properties of these cells and a decrease in their viability (Figure 2 and Figure 3). These indicators tended to decrease after exposure to 50 μg of protein, while a significant cytotoxic effect was observed when cells were exposed to 100 and 200 μg of the studied protein, with a decrease in the proliferative properties of cells by 40 and 70–75%, respectively. We performed a similar series of experiments using human embryonic kidney (HEK 293) cells as an example (Figure 2B), but no significant changes in the proliferative activity of these cells after exposure to hSELENOM at concentrations of 50 and 100 μg were observed. A marked decrease (approximately 20–25%) in the viability of HEK 293 cells was observed only after they were treated with a high concentration (200 μg) of the protein under study.

This selectivity of recombinant hSELENOM towards cancer cells is a very important property and can be explained by the fact that the microtumor environment (in the nutrient medium in this case) most likely contains a large amount of reduced thiols. On the other hand, many cancer cells are known to have elevated levels of reduced glutathione [41]. hSELENOM penetrates into cells, most likely via actin-dependent endocytosis as shown in this work (Figure 8), and interacts with reduced glutathione due to the presence of the CXCS active center (where C is cysteine and X is any amino acid). Thus, the ratio of oxidized/reduced glutathione is either 1:1 or shifts towards the oxidized form and is 3:1. These changes in the redox status can significantly affect the correct folding of proteins in the lumen of the endoplasmic reticulum, which leads to the accumulation of misfolded proteins and is the cause of ER stress activation. In the case of prolonged ER stress, the cell cannot cope and goes into apoptosis, which was demonstrated in this work. Interestingly, with a decrease in the activity of this selenoprotein in the same A-172 cell line, no significant changes were observed in the proliferative properties and viability of these cancer cells [13].

Furthermore, here we have shown (Figure 4 and Figure 5) that treatment of cancer cells with hSELENOM resulted in the increased expression of a key marker of one of the three known UPR (unfolding protein response) signaling pathways—IRE1α. It is known that during the growth of proteins with incorrect folding in the lumen of the ER, the cell responds to this situation by activating UPR signaling pathways. In this case, we observed that hSELENOM caused an increase in the expression of the spliced form of the XBP1s transcription factor, which may be due to the activation of both IRE1α and ATF-6 UPR signaling pathways, since the active form of the transcription factor ATF-6 can bind to the stress-sensitive element CCAAT (N)9CCACG in the XBP1 sequence and enhance its expression [42,43]. This transcription factor controls the proliferation of ER structures, protein maturation, and its own release from the ER. XBP1 also promotes the removal of proteins with irreversibly disrupted conformations from the ER [44]. However, real-time PCR did not reveal any significant change in ATF-6 mRNA expression; therefore, the version of activation of the IRE1α-signaling UPR pathway after 24 h treatment of A-172 cells with recombinant hSELENOM seems to be the most convincing mechanism. The activation of ER stress can be supported by the data on increased expression of genes such as CHOP, GADD34, PUMA, and CAS-4 (Figure 3 and Figure 4). CHOP (CCAAT/enhancer-binding protein-homologous protein) is usually activated during ER stress [45,46,47,48]. Activated CHOP induces a number of genes encoding proteins involved in apoptosis, such as GADD34 (growth retardation protein induced by DNA damage) and PUMA (p53-regulated modulator of apoptosis), which is activated by p53 during ER stress.

Enhancement of mRNA expression of the gene encoding caspase-4 is known to be possible in two ways. First, it was found that TRAF2 (a factor associated with the TNF receptor) interacts with pro-caspase-12 under ER stress [49], which activates caspase-9, which forms apoptosomes that are necessary for the activation of effector caspase-3 [50,51,52]. Caspase-12 has been identified in rodents, while caspase-4 presumably plays its role in humans [53]. Secondly, the activation of caspase-12 under conditions of ER stress seems to be mediated by the Ca^2+^ signaling pathway. When ER stress occurs, conformational changes and/or oligomerization of pro-apoptotic Bax and Bak proteins (proteins belonging to the Bcl-2 family) occur on the ER membrane, which leads to damage to calcium depots in the ER and the release of Ca^2+^ into the cytosol [54,55]. An increased flow of Ca^2+^ activates m-calpain, which is a member of the family of Ca^2+^-dependent cysteine proteases [51]. Calpain, in turn, cleaves pro-caspase-12 to caspase-12, which leads to the activation of apoptosis [56,57,58].

The second scenario is supported by the data obtained by us in the course of this work showing that recombinant hSELENOM can dose-dependently deplete the Ca^2+^ pool of the ER. Our experiments showed that the application of hSELENOM in an acute experiment did not cause an increase in Ca^2+^ ions in the cytosol of A-172 cells (Figure 7). We observed the effect of Ca^2+^ depletion of the endoplasmic reticulum pool 24 h after incubation of A-172 cancer cells with hSELENOM. It can be assumed that endocytosis of hSELENOM into cancer cells led to the leakage of Ca^2+^ ions from the ER and a gradual increase in [Ca^2+^]_i_. This increase in Ca^2+^ ions in the cytosol leads to the activation of m-calpain which in turn leads to the activation of CAS-4 and the induction of apoptosis. It is known that leakage of Ca^2+^ ions from the ER pool can activate apoptosis [59,60]. This cascade of events can lead to disruption of Ca^2+^ homeostasis of the cells, and a reduced baseline level of [Ca^2+^]_i_ after treatment of glioblastoma A-172 cells with hSELENOM.

Interestingly, the treatment of human glioblastoma cells with recombinant hSELENOM led to an increase in SELENOM mRNA expression. It is unlikely that the recombinant protein may be involved in the regulation of the mRNA expression of the native protein. Most likely, the increasing oxidative and ER stress in cancer cells caused by the action of recombinant hSELENOM activates the expression of the native protein gene, which was repeatedly shown in our early works [16,17,18].

On the other hand, when the same cancer cell line was treated with hSELENOM-KD, an increase in the expression of the CHOP, GADD34, and PUMA genes was also observed, but no increase in the expression of the CAS-4 mRNA and key UPR markers was observed [13]. This may indicate that the activation of ER stress is caused directly by overexpression of either the native hSELENOM protein or the recombinant hSELENOM.

Thus, in this work, it was reliably shown that incubation of human glioblastoma cells with the recombinant cysteine homologue of the human selenoprotein hSELENOM leads to a decrease in the proliferative activity of these cancer cells and activation of the IRE1α signaling pathway UPR. Activation of apoptosis was also observed, most likely through disturbance of calcium homeostasis caused by depletion of the ER depot, activation of m-calpain, and subsequent activation of CAS-4. 

## 4. Materials and Methods

### 4.1. Cell Culture and Bacterial Strains

The human glioblastoma (A-172) and human embryonic kidney (HEK 293) cell lines used in this study were purchased from ATCC, Manassas, VA, USA. The cells were grown in DMEM nutrient medium supplemented with 10% serum and antibiotics.

The top 10 derivatives of strain DH10B were purchased from Invitrogen, (Thermo Fisher Scientific, Waltham, MA, USA). Rosetta (DE3)pLysS, a derivative of strain BL21(DE3), contains the pLysE plasmid encoding T7 lysozyme, a natural inhibitor of T7 RNA pol, was purchased from Novagen (Darmstadt, Germany).

### 4.2. Isolation of RNA and Reverse Transcription

Isolation of total RNA from cells was performed using the RNA extract reagent (Evrogen, Moscow, Russia) according to the manufacturer’s protocol. To prevent contamination of genomic DNA, RNA samples were treated with DNase I at 37 °C for 1 h, after which the enzyme was inactivated by adding 50 mM EDTA and heating to 60 °C for 10 min. The amount of total RNA used in the reverse transcription reaction averaged 1 μg. The reverse transcription reaction was carried out according to the protocol and using a kit for the synthesis of the first strand cDNA (Evrogen, Moscow, Russia). The reaction was carried out in the presence of oligo(dT) primers.

### 4.3. Cloning and Site-Directed Mutagenesis

To insert the open reading frame (ORF) of the gene encoding human SELENOM (hSELENOM) (NM_080430.3) into the pET23b(+) plasmid (Novagene, Sacramento, CA, USA) to express hSELENOM protein in a bacterial system, a routine cloning method was performed using restriction and ligation enzymes (Thermo Fisher Scientific, USA). Preliminarily, total RNA was isolated from human embryonic kidney (HEK 293) cell line and used in a reverse transcription reaction. RNA isolation and reverse transcription were performed as described above. Restriction was carried out using EcoR I and SalI restriction enzymes for 3 h at 37 °C, after which the cut PCR products were purified from restriction enzymes according to the Evrogen protocol. Next, the ligation reaction was performed using T4 DNA ligase (Thermo Fisher Scientific, Waltham, MA, USA) for 2 h at room temperature. Ligation was carried out at 4 °C for 16 h or 3 h at room temperature, and then the ligation mixture was used to transform the bacterial strain Top 10. Site-directed mutagenesis was performed by PCR using overlapping primers, the sequences of which are shown in Table 1.

### 4.4. Isolation and Purification of Recombinant hSELENOM

Purification of the recombinant hSELENOM was carried out using affinity chromatography on nickel agarose (Thermo Fisher Scientific, USA).The procedure for isolating and purifying the recombinant protein was carried out under native conditions, in which the cells were resuspended in buffer (50 mM NaH_2_PO_4_, 300 mM NaCl, 10 mM imidazole, and 1 mM PMSF, pH 8.0). The cells were lysed using an ultrasonic disintegrator at a frequency of 22 kHz for 10 min at 4 °C, and then centrifuged at 10,000 rpm for 20 min. After that, the amount of protein in the soluble fraction and sediment was estimated by PAGE electrophoresis. The soluble protein fraction was filtered through a 0.45 μM filter to remove cell debris and applied to Poly–Prep Chromatography Columns (Bio–Rad, Hercules, CA, USA) containing nickel agarose. Protein binding to nickel agarose was carried out at 4 °C for 3 h, after which it was thoroughly washed with a buffer (50 mM NaH_2_PO_4_, 300 mM NaCl, 20 mM imidazole, pH 8.0); then the protein was eluted from the column by incubation with the same buffer, but the content of imidazole in it was 150–250 mM. The recombinant proteins were analyzed by PAGE electrophoresis using 10 or 12% polyacrylamide resolving gels. For further work with the purified recombinant protein, stepwise dialysis was performed to reduce the concentration of imidazole in the elution buffer. If the protein was eluted with the addition of large volumes of buffer, then it was further concentrated using special membranes for an Amicon Ultra 4–50 kDa centrifuge filter (Merk Millipore, Darmstadt, Germany).

### 4.5. Real-Time PCR

The real-time PCR reaction was performed using the qPCRmix–HS SYBR mixture (Evrogen, Moscow, Russia) according to the manufacturer’s protocol. The removal of fluorescence occurred at the stage of elongation; the melting curve was measured from 60 to 95 °C with a step of 0.3 °C. The results were expressed as 2^−∆(∆Ct)^, where ∆(∆Ct) is the difference between the ∆Ct values between the gene under study and a housekeeping gene. The sequences of all primers used in the real-time PCRs are presented in Table 2. The specificity of the synthesized PCR products was checked by fragment electrophoresis in 2–3% agarose gels.

### 4.6. Western Blotting

Samples were prepared by resuspending cells in a buffer containing 100 mM Tris-HCl, pH 8.0, 0.15 mM NaCl, 1 mM EDTA, and 1 mM PMSF, at 4 °C and analyzed by PAG electrophoresis in 12.5% separating gels. Next, the proteins were electrotransferred from the gel onto a PVDF membrane, which was blocked with 5% skim milk for 3 h at room temperature, after which the membrane was washed three times with 1× PBST buffer (pH = 7.4). The immunodetection procedure was performed using primary antibodies against the label or protein of interest and secondary antibodies conjugated with horseradish peroxidase. To do this, the membranes were incubated with each of the antibodies for 2–3 h at room temperature. The Western blotting results were analyzed by DAB staining (0.05% DAB in TBS + 10 µL 30% hydrogen peroxide). We used the following primary and secondary commercial antibodies purchased from Abcam (Cambridge, UK) and Invitrogen (Thermo Fisher Scientific, Waltham, MA, USA): anti-GAPDH (#437000, Invitrogen), anti-SELENOM (#PA5-72639, Invitrogen), anti-XBP1 (#PA5-27650, Invitrogen), anti-Cas-4 (#EPR20921-83, Abcam), anti-CHOP (#MA1-250, Invitrogen), and horseradish peroxidase-conjugated secondary (#656120, Invitrogen) antibodies.

### 4.7. Registration of Changes in Cytosolic Ca^2+^

Registration of the dynamics of the cytosolic Ca^2+^ levels in cells was carried out using the fluorescent calcium probe Fura-2AM. The cells were loaded with a probe that was diluted with Hanks’ balanced salt solution (HBSS) (156 mM NaCl, 3 mM KCl, 2 mM MgSO_4_, 1.25 mM KH_2_PO_4_, 2 mM CaCl_2_, 10 mM glucose and 10 mM HEPES, pH 7.4) at 37 °C for 40 min. Further washing was carried out for 15 min. The changes in [Ca^2+^]_i_ were recorded using an Axiovert 200M inverted motorized fluorescence microscope (Carl Zeiss Microscopy GmbH, Jena, Germany). The reagents were added and washed with a perfusion system providing a perfusion rate of 15 mL/min. The Fura-2 was excited and recorded using an HCX PL APO 20.0 × 0.70 IMM UV lens with a refractive index of 1.52. The resulting time-lapse series of images were processed using ImageJ software (Java 1.6.0_12, RRID: SCR_003070, LOCI, University of Wisconsin, Madison, WI, USA). Background fluorescence was subtracted frame by frame using ImageJ’s Math Subtract plugin. All experiments were carried out at a temperature of 28–30 °C.

### 4.8. MTT Analysis and Apoptosis Detection Assay

MTT analysis was performed using the MTT kit (Abcam, UK) according to the manufacturer’s protocol. The optical density of the solutions in each test well was carried out at OD = 570 nm using a tablet reader. This procedure was repeated three times

An apoptosis/necrosis reagent kit (#ab176749, Abcam) was used to detect healthy cells and cells with signs of apoptosis. To detect cells with signs of apoptotic death, the Apopxin Green Indicator was used and they were visualized using a fluorescent microscope using the FITC channel (Ex/Em = 490/525 nm). CytoCalcein 450 was used to detect healthy cells and the cells were visualized with the violet channel (Ex/Em = 405/450 nm).

### 4.9. Statistical Analysis

Protein expression was quantified using ImageJ software (Java 1.6.0_12, RRID: SCR_003070, LOCI, University of Wisconsin, Madison, WI, USA). Origin v8.5 (Microcal Software Inc., Northampton, MA, USA) and Prism 5 (GraphPad Software, La Jolla, CA, USA) were used for plotting the data and statistical processing. The significance of differences between groups and within experimental groups was determined using Student’s *t*-test. Differences were considered significant at *p* < 0.05. *** *p* < 0.001, ** *p* < 0.01, * *p* < 0.05, n/s—differences are not significant.

## 5. Conclusions

In the work, a recombinant hSELENOM, in which the TGA codon was replaced by TGT, in preparative amounts was obtained. It has been shown that incubation of human glioblastoma cells (A-172 line) with 50, 100, and 200 μg of hSELENOM for 24 h contributed to a dose-dependent decrease in the proliferative properties of these cells, while the cytotoxic effect of this protein was practically absent when non-cancer human embryonic kidney cells (HEK 293) were treated. Recombinant hSELENOM penetrated into A-172 cells by actin-dependent endocytosis, inducing apoptosis of the cells, causing an increase in the expression of mRNA of pro-apoptotic genes and the UPR IRE1α signaling pathway, and also depleting the ER pool of Ca^2+^.

## Figures and Tables

**Table 1:** Oligonucleotide Sequences Used for SELENOM ORF Amplification and Single Nucleotide Substitution.

Gene Name	Forward Primer 5′– > 3′	Reverse Primer 5′– > 3′
TGA– > TGT	GTAGAGACCTGCGGGGGATGTCAGCTGAACCGCC	GCTGACATCCCCCGCAGGTCTCTACCCG
Cloning primers	TACAGAATTCATGAGCCTCCTGTTGCCTCCG	CGTCGACCTACAGGTCAGCGTGGTCCGAAG

**Table 2:** Primers for the synthesis of human mRNA fragments.

Gene Name	Forward Primer 5′– > 3′	Reverse Primer 5′– > 3′
GAPDH	ACATCGCTCAGACACCATG	GCCAGTGAGCTTCCCGTT
SELENOT	TCTCCTAGTGGCGGCGTC	GTCTATATATTGGTTGAGGGAGG
SELENOM	AGCCTCCTGTTGCCTCCGC	AGGTCAGCGTGGTCCGAAG
SELENOF	TACGGTTGTTGTTGGCGAC	CAAATTGTGCTTCCTCCTGAC
SELENOK	TTTACATCTCGAACGGACAAG	CAGCCTTCCACTTCTTGATG
SELENOS	TGGGACAGCATGCAAGAAG	GCGTCCAGGTCTCCAGG
SELENON	TGATCTGCCTGCCCAATG	TCAGGAACTGCATGTAGGTGG
DIO2	AGCTTCCTCCTCGATGCC	AAAGGAGGTCAAGTGGCTG
CHOP	GCTCTGATTGACCGAATGG	TCTGGGAAAGGTGGGTAGTG
GADD34	CTCCGAGAAGGTCACTGTCC	GACGAGCGGGAAGGTGTGG
PUMA	CAGATATGCGCCCAGAGAT	CCATTCGTGGGTGGTCTTC
BIM	GGACGACCTCAACGCACAGTACGAG	GTAAGGGCAGGAGTCCCA
CAS-3	GCATTGAGACAGACAGTGGTG	AATAGAGTTCTTTTGTGAGCATG
CAS-4	CACGCCTGGCTCTCATCATA	TAGCAAATGCCCTCAGCG
BAX	GGGCTGGACATTGGACTTC	AACACAGTCCAAGGCAGCTG
BAK	GAGAGTGGCATCAATTGGGG	CAGCCACCCCTCTGTGCAATCCA
BCL-2	GGTGAACTGGGGGAGGATTG	AGCCAGGAGAAATCAAACAGAG
ATF-4	GTGTTCTCTGTGGGTCTGCC	GACCCTTTTCTTCCCCCTTG
ATF-6	AACCCTAGTGTGAGCCCTGC	GTTCAGAGCACCCTGAAGA
XBPu	ACTCAGACTACGTGCACCTC	GTCAATACCGCCAGAATCC
XBPs	CTGAGTCCGCAGCGGTGCAGG	GGTCCAAGTTGTCCAGAATG

## Data Availability

The data presented in this study are available upon request from the corresponding author.

## Supplementary Materials

The following are available online at https://www.mdpi.com/article/10.3390/ijms24076469/s1, Original western blotting of A-172 cells after 24 h of exposure to 100 μg of recombinant hSELENOM (1 μg/μL).

## Abbreviations

ATF-4, transcription factor with a bZIP leucine zipper domain, a member of the ATF/CREB b-ZIP family of proteins; BAK, pro-apoptotic protein of the BCL-2 family, homologous antagonist/killer of BCL-2; BAX, pro-apoptotic protein of the BCL-2 family, BCL-2-like protein 4; BCL-2, B-cell lymphoma anti-apoptotic protein 2; BIM, pro-apoptotic protein of the BCL-2 family interacting with anti-apoptotic proteins BCL-2 and BCL-XL; CAS-3, caspase 3; CHOP, pro-apoptotic transcription factor, member of the DNA-binding transcription factor family of CAAT/enhancer-binding proteins; DIO2, deiodinase 2; ER, endoplasmic reticulum; GADD34, growth retardation and DNA damage gene-34; IRE1α, α-isoform of inositol-dependent enzyme type 1; PUMA, p53 up-modulating apoptosis; SELENO, selenoprotein; UPR, cellular response to an increase in misfolded proteins; XBP1, X-box binding protein 1.
