# Supplementary figures and images for: Molecular Mechanisms of the Cytotoxic Effect of Recombinant Selenoprotein SELENOM on Human Glioblastoma Cells
Source: Int J Mol Sci. 2023 Mar 30;24(7):6469. doi: 10.3390/ijms24076469 (PMC10094712; doi:10.3390/ijms24076469)

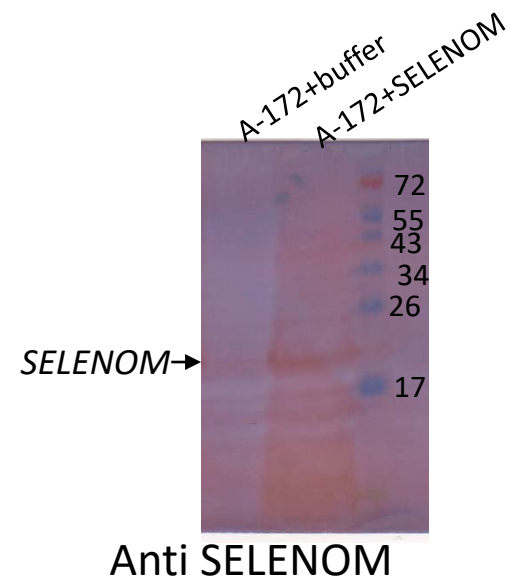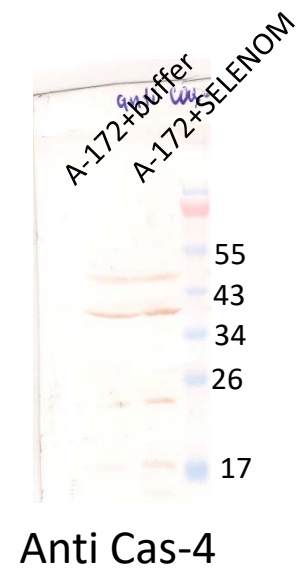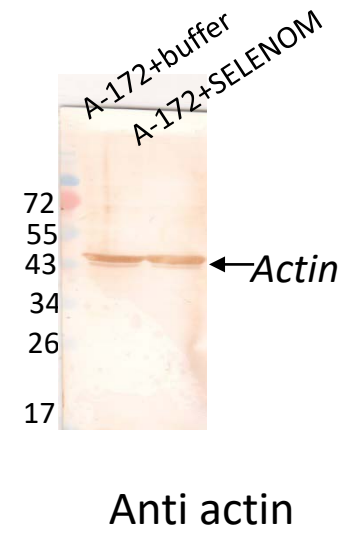

Supplement: Supplementary file 1 [file ijms-24-06469-s001.zip › ijms-2268909-SI.pdf]
